# Clinical Epidemiology of Lymphatic Filariasis and Community Practices and Perceptions Amongst the Ado People of Benue State, Nigeria

**Published:** 2011

**Authors:** Edward Agbo Omudu, Jennifer Ochanya Ochoga

**Affiliations:** Department of Biological Sciences, Benue State University, P. M. B. 102119, Makurdi, Nigeria

**Keywords:** Lymphatic filariasis, Lymphoedema, Hydrocoele, Circulating Filarial Antigen, Benue State, Nigeria

## Abstract

As part of efforts to initiate lymphatic filariasis elimination activities in Benue State, this study employed the use of lymphatic filariasis-related clinical signs as rapid diagnostic features, immunochromatographic card test (ICT) to detect circulating filarial antigen (CFA) and questionnaire to investigate community perceptions and beliefs. 81 (32.6%) out of the 248 persons were positive for circulating filarial antigen (CFA). Infection rates denoted by CFA ranged from 41 (46.1%) in Uffia to 1(6.6%) in Ijigbam districts. Distribution of community ICT prevalence showed a significant variation (X^2^, P < 0.05). The prevalence of clinical signs and/or symptoms in the communities also showed significant variations (X^2^, P < 0.05). Community hydrocoele prevalence ranged from 8 (9.0%) in Uffia to 1(6.6%) in Ijigbam. The overall hydrocoele prevalence was 21 (8.5%), while the overall lymphoedema prevalence was 16 (6.4%) and women accounted for 14 (87.5%) of persons with swollen limbs. Only about 14 (15.9%) of unaffected respondents knew that lymphatic filariasis is transmitted through mosquito bites, this differ significantly from affected respondents 10 (66.6%) (X^2^, P < 0.05). The communities' capacity to protect themselves is hindered by a lack of understanding of the true cause, symptoms, transmission route and prevention of the disease. Our study demonstrates the need for the development of health education programmes that will enable people to protect themselves against mosquito bites. As Nigeria commence her lymphatic filariasis elimination programmes, there is an urgent need to develop morbidity management activities that will alleviate the burden of patients.

## Introduction

Lymphatic filariasis is a tropical disease caused by the parasitic filarid nematode worm *Wuchereria bancrofti*. About 128 million people in 73 countries are infected globally with an estimated 1.1 billion at risk of infection (Addiss and Brady, 2007). The disease is the second leading cause of permanent and long-term disability in the world, inflicting serious public health and socio-economic problem in endemic communities (Terranella et al., 2006). At least 40 million people are affected in Africa (Anosike et al., 2005). The disease is usually seen among the poorest of the poor, and for many years has had as very low public health rating in the priorities of most of the countries where it is prevalent. The visible manifestations of the disease are severe and disfiguring, it has been reported that one third of infected individuals present with overt clinical manifestations: lymphoedema and elephantiasis of the limbs, or genitals, hydrocoele, chyluria, or recurrent infections associated with damaged lymphatic (Sherchand et al., 2003). The acute attacks of adenolymphangitis (ADL) are characterized by fever, chills, local warmth and inflammation of the inguinal node. Patients are usually incapacitated for 4–7 days while the attack lasts. The swelling later becomes permanent in the form of lymphoedema and at times there is dysfunction of the genital lymphatic that leads to hydrocoeles (Person et al., 2006).

Lymphatic filariasis is prevalent in Nigeria and widespread, Nigeria is believed to be the third most endemic country in the world after India and Indonesia (Eigege et al., 2002). Studies in Nigeria have reported prevalence rates ranging from 6% – 47% (Anosike et al., 2005, Eigege et al., 2002, Udoidung et al., 2008, Braide et al., 2003, Nwoke et al., 2006, Badaki and Akogun 2000, Mba and Njoku 2000, Omudu and Okafor 2007). A lot more epidemiological information is however needed on the distribution, clinical signs, burden and impact of the disease on individuals. Several researchers have also highlighted the dearth of sociocultural information on the local beliefs, perceptions and behaviours towards the disease (Addiss and Brady 2007). This paucity of sociocultural data is a common feature of lymphatic filariasis and other neglected tropical diseases. Gyapong et al. (1996) have argued that the lack of information and understanding of lymphatic filariasis sociocultural consequences have led to a gross underestimation of its impact in rural communities. Lack of the correct disease etiology may increase risk, impede progress of intervention and become obstacles to health seeking behaviour.

The global effort to eliminate lymphatic filariasis as a public health problem focuses on Mass Drug Administration (MDA) following community diagnosis using the prevalence of hydrocoele and lymphoedema as rapid diagnostic features (Eigege et al., 2002, Nwoke et al., 2006, Gyapong et al., 1998). This paper presents findings from a comparative study using lymphatic filariasis-related clinical signs as rapid diagnostic feature and serological diagnosis using the immunochromatographic card test to detect circulating filarial antigen (CFA), and community perceptions and beliefs on these disease manifestations. The study intends to provide much needed epidemiological data that will guide in identifying high risk communities and develop evidence-based community education strategies.

## Materials and Methods

### Description of Study Area

Benue state is one of the 36 states in the Federal Republic of Nigeria, a tropical country on the west coast of Africa. The state derives its name from River Benue the second largest river in the country and is located in the central region of the country where it lies between latitude 7° 13 and 7° 49 longitude 8° 15 and 8° 42. The state covers an area of about 34, 059 square kilometers with a population of over 4.2 million people (National Population Commission, 2007). Majority of the inhabitants live in rural agricultural areas and engage in peasant agriculture, the state's reputation as the food basket of the nation is being seriously jeopardized by the socio-economic consequences of parasitic diseases. Ado Local Government Area (LGA) where the study was conducted is one of the twenty three (23) LGAs of the state with a population of 178.882 (National Population Commission, 2007). The Local Government is made up of five major districts, Agila, Igumale, Ulayi, Ijigbam and Utonkon. The LGA is located at the southern part of Benue State (Fiure 1) and has boundaries with Ebonyi and Enugu States. The inhabitants are mainly peasant farmers and live in small farming settlements. The Ado and Okpokwu rivers are the predominant rivers and provide breeding ground for vectors of some filarial diseases. The study was conducted from February – November 2009.

### Ethical Clearance

This survey received ethical clearance from the State Ministry of Health and the proposal was approved by the Postgraduate Research Committee of the University of Nigeria, Nsukka. Informed consent was obtained from all the participants after the explanation of the procedures and the likely benefits of the study. After explaining the purpose of the study to the village chiefs and traditional leadership councils and obtaining their permission and consent, all participating adults (16 years of age and older) were asked to gather at the village Primary Health Care (PHC) Centre and randomly selected. Before clinical examination and testing could be carried, the objectives of the survey were explained in local language and each consenting individual provided demographic data. The participants were assigned identification numbers and their names, age, occupation and marital status were taken.

### Search for Hydrocoele and Lymphoedema

After obtaining demographic information (age, occupation, marital status, sex), participants were asked to partially disrobe and a local government health officer trained on the diagnosis of the various manifestations of filariasis including hydrocoele and lymphoedema. Hydrocoele was diagnosed based on the finding of a non-tender, soft, fluid-filled mass bigger than the size of an orange. Hydrocoele was distinguished from inguinal hernia (which would change with cough or straining, show an inguinal swelling at the internal ring) as described Eigege et al. (2005). Clinical examination also involved the search for lymphoedema which was easier to conduct than the search for hydrocoele. Participants were simply asked to lift up their clothing to expose their legs. Swollen limbs and leopard skin were observed and noted (Anosike et al., 2005; Eigege et al., 2002; Nwoke et al., 2006, Omudu and Okafor 2007). Dermal manifestations associated with Onchocerciasis like leopard skins, palpable nodules, onchocercal dermatitis (creeping eruption) and crawling sensation were also recorded during the clinical examination.

### Immunochromatographic card test procedure

After collecting the demographic data, ICT testing was performed following kit instructions (NOW, ICT filariasis kits; Binax, Portland, ME, USA). The patient's left index finger was cleaned with methylated spirit and then punctured using a sterile lancet. The initial sample of blood was removed using a cotton swab, and sufficient fresh blood was then obtained to fill a 100-ql capillary tube. The blood was then transferred from the capillary tube to the pad on the ICT test kit card and then sealed. The results of each ICT test card were read after 15 mins. A positive result was when two pink lines appeared on the card's window, and a negative result was when a single line was seen. Test results with the individual's identification code were recorded on the patients' diagnostic data sheet.

### Interviews and Questionnaire Administration

Interviews, using semi-structured questionnaire were conducted with selected individuals from all the districts to gather descriptive information on villagers' knowledge and beliefs about the cause, mode of transmission and how to prevent the disease. Based on the descriptive information, a structured questionnaire was developed. It included 19 questions on villagers' awareness, knowledge beliefs and health seeking behaviour in relation to filariasis. Questionnaire was administered to both affected and unaffected residents of the community. A total of 103 volunteers comprising 15 persons with visible clinical signs of lymphatic filariasis and 88 persons without visible signs participated in the questionnaire aspect of the study.

### Data Analysis

The quantitative epidemiological data was entered into Epi-info software (version 6.0: CDC Atlanta, USA) and was analysed in percentages using chi-square to test significance. The questionnaire data was analysed using the SPSS package.

## Results

Two hundred and forty eight (248) persons from 7 communities situated in Ado LGA of Benue State, Nigeria, were clinically examined for manifestations of filariases and detection of circulating *W. bancrofti* using the ICT card tests. 81 (32.6%) out of the 248 persons were positive for circulating filarial antigen (CFA). Infection rates of CFA ranged from 41 (46.1%) in Uffia to 1(6.6%) in Ijigbam (Table 1). Distribution of community ICT prevalence showed a significant variation (X^2^ P < 0.05). The prevalence of clinical signs/symptoms in the communities also showed significant variations (X^2^ P < 0.05). Community hydrocoele prevalence ranged from 8 (9.0%) in Uffia to 1(6.6%) in Ijigbam, the overall hydrocoele prevalence was 21 (8.5%). While the overall lymphoedema prevalence was 16 (6.4%) and women accounted for 14 (87.5%) of persons with swollen limb. Clinical manifestations associated with Onchocerciasis were also prevalent in the communities. These include leopard skin which had overall prevalence of 19 (7.6%), palpable nodules 43 (17.3%), Dermatitis 105(42.3%) skin rashes and itching and/or crawling sensation 112(45.2%) (Table 1). While dermatitis and a crawling sensation appeared much earlier in life, hydrocoele, leopard skin and lymphoedema showed up from the age range of 40 and above (Table 2).

**Table 1:** Community prevalence of clinical manifestations of filariases and lymphatic filariasis antigen as detected by ICT card test.

Communities	Number examined	Number with hydrocoele	Number with lymphoedema	Number with leopard skin	ICT positive	Number with palpable module	Number with skin eruption	Number with crawling sensation	Number who had taken Ivermectin
Uffia	89	8(9.0)	2(2.2)	4(4.5)	42(46.1)	13(14.6)	34(38.2)	31(34.8)	44(49.4)
Apa	43	2(4.6)	4(9.3)	2(4.6)	14(32.5)	3(6.9)	2(48.8)	21(48.8)	23(53.4)
Agila	25	1(4.0)	2(8.0)	3(12.0)	6(24.0)	5(20.0)	11(44.0)	11(44.0)	4(16.0)
Ezza	38	4(10.5)	2(5.3)	3(7.9)	13(34.2)	7(44.7)	18(47.3)	15(39.5)	11(29.0)
Igumale	20	3(15.0)	2(10.0)	4(20.0)	5(25.0)	5(25.0)	8(40.0)	4(70.0)	5(25.0)
Ijigbam	15	1(6.6)	1(6.6)	1(6.6)	1(6.6)	6(40.0)	3(20.0)	9(60.0)	1(6.6)
Ulayi	18	2(11.1)	3(16.6)	2(11.1)	-	4(22.2)	10(55.5)	11(61.1)	8(44.9)

**Total**	**248**	**21(8.5)**	**16(6.4)**	**19(7.6)**	**81(32.6)**	**43(17.3)**	**105(42.3)**	**112(45.2)**	**106(42.7)**

Figures in parenthesis are percentages (%)

**Table 2:** Age-related prevalence of clinical manifestations of filariasis and detection of lymphatic filariasis antigen using ICT card test.

Age group	Number examined	ICT positive	Number with hydrocoele	Number with lymphoedema	No with leopard skin	Number with palpable module	Number with skin eruption	Number with crawling sensation	Number Ivermectin Treatment
1 – 10	3	−(0.0)	−(0.0)	−(0.0)	−(0.0)	−(0.0)	1(33.3)	1(33.3)	1(33.3)
11 – 20	12	4(33.3)	−(0.0)	−(0.0)	−(0.0)	−2(16.6)	2(16.6)	3(25.0)	4(33.3)
21 – 30	30	9(30.0)	1(3.3)	1(3.3)	−(0.0)	6(20.0)	16(53.3)	20(66.6)	21(70.0)
31 – 40	73	28(38.3)	5(6.8)	2(2.7)	2(2.7)	13(17.8)	25(34.2)	20(27.3)	19(26.0)
41 – 50	71	21(29.5)	5 (7.0)	3(4.2)	9(12.6)	13(18.3)	34(47.8)	25(35.2)	20(28.1)
51 – 60	39	16(41.0)	7(17.9)	4(10.2)	6(15.3)	6(15.3)	18(46.1)	23(58.9)	27(69.2)
61 – 70	14	2(14.2)	1(7.1)	3(21.4)	1(7.1)	3(21.4)	6(42.8)	14(100.0)	8(57.1)
70 – above	6	1(16.6)	3(50.0)	3(50.0)	1(16.6)	−(0.0)	3(50.0)	6(100.0)	6(100.0)

**Total**	**248**	**81(32.6)**	**21(8.5)**	**16(6.4)**	**19(7.6)**	**4.3(17.3)**	**105(42.2)**	**112(45.2)**	**106(42.7)**

Figures in parenthesis are percentages (%)

### Knowledge about the cause, transmission and prevention

Only about 14 (15.9%) of unaffected respondents knew that lymphatic filariasis is caused through mosquito bites, this differ significantly from affected respondents 10 (66.6%) (X^2^, P < 0.05) (Table 4). Half of the unaffected respondents attributed the cause of the disease to stepping on charms. The other major responses on cause of the disease include lack of personal hygiene and sexual intercourse with infected persons. The respondents' perception of the cause of the disease is consistent with the beliefs on mode of transmission. Only 18 (20.4%) of unaffected respondents cited mosquito bite as route of transmission and this varied significantly from 8 (53.3%) affected respondents (X^2^ P < 0.05). 50 (56.8%) and 56 (63.6%) of unaffected respondents again attributed the mode of transmission of the disease to witchcraft and stepping on charms respectively (Table 5). While 14 (93.3%) of affected respondents knew that protection from mosquito bites can prevent filariasis, only 15 (17.0%) of unaffected respondents shared the same opinion. Other means of prevention of lymphatic filariasis as cited by 20 (22.7%) of unaffected respondents include avoidance of body contact and sexual intercourse with infected persons (Table 6). On the whole, affected respondents were more knowledgeable on the correct etiology of the disease than and unaffected respondents.

**Table 4:** Respondents knowledge on causes of lymphatic filariasis

Causes	Affected n =15 Yes (%)	Unaffected n = 88 Yes (%)
Working in the sun	2 (13.3)	2 (2.3)
Walking long distance	5 (33.3)	5 (5.7)
Sexual intercourse	1 (6.6)	13 (14.7)
Stepping on charm	5 (33.3)	44 (50.0)
Contaminated food	2 (13.3)	13 (14.7)
Lack of personal hygiene	8 (53.3)	43 (49.0)
Fever	- (0.0)	9 (10.2)
Mosquito bites	10 (66.6)	14 (15.9)

**Table 5:** Respondents beliefs on mode of transmission of lymphatic filariasis

Mode of transmission	Affected n =15 Yes (%)	Unaffected n = 88 Yes (%)
Sexual intercourse	1 (6.6)	18 (20.4)
Body contact	4 (26.6)	17 (19.3)
Witchcraft	3 (20.0)	50 (56.8)
Food poisoning	1 (6.6)	29 (32.9)
Mosquito bites	8 (53.3)	18 (20.4)
Stepping on charm	2 (13.3)	56 (63.6)
Inheritance	2 (13.3)	17 (19.3)

**Table 6:** Respondents belief on the prevention of lymphatic filariasis

Preventive measures	Affected n =15 Yes (%)	Unaffected n =88 Yes (%)
Avoid sexual intercourse with affected person	5 (33.3)	20 (22.7)
Avoid body contact with affected person	1 (6.6)	20 (22.7)
Sacrifice to appease gods	5 (33.3)	34 (38.6)
Good personal hygiene	6 (40.0)	50 (56.8)
Avoid mosquito bites	14 (93.3)	15 (17.0)
Avoid eating with affected person	1 (6.6)	2 (2.3)

### Perceived socioeconomic and psychological implications of lymphatic filariasis

Social and psychological impact of the disease include personal discomfort in public and inability to engage in income generating activities, both affected and unaffected respondents generally agreed on these consequences. However, there was a significant difference between affected and unaffected respondents in the belief that the disease manifestations affect sexual relation with spouse. While 60 (68.1%) of unaffected persons were of the opinion that the disease manifestations affected sexual relations with spouse. Only 4 (26.6%) of affected persons shared the same opinion (X^2^ P < 0.05) (Table 7). Lymphatic filariasis — related health expenditure seem to be underrated by unaffected persons, their estimate of monthly health seeking expenditure by patients were significantly lower that those of affected respondents. 9 (60.0%) of affected respondents estimated the monthly health seeking expenditure at between N2000 – N3000. only 25 (28.4%) of unaffected respondent corroborated this estimate (Table 8). Convenience and affordability were key factors influencing the choice of health provider by infected persons (Table 8). However unaffected respondents believed that the choice of health provider to patronize is influenced by family decision.

**Table 7:** Respondents perception on some socio-economic and psychological consequences of lymphatic filariasis

Consequences	Affected n =15 Yes (%)	Unaffected n =88 Yes (%)
Personally uncomfortable in public	14 (93.3)	77 (87.5)
Affects work and income	14 (93.3)	83 (94.3)
Affects sexual relation with spouse	4 (26.6)	60 (68.1)
Hinder marriage prospects of other members of family	3 (20.0)	52 (59.1)
Spouse desertion and divorce	3 (20.0)	39 (44.3)
Suspicion of infidelity	2 (13.3)	30 (34.1)

**Table 8:** Respondents perception of the monthly lymphatic filariasis related health expenditure and factors that influence choice of health providers.

Expenditure	Affected n =15 Yes (%)	Unaffected n =88 Yes (%)
Below N500 monthly	7 (38.8)	12 (13.6)
Between N500 – N1000	5 (33.3)	20 (22.7)
Between N1000 – N2000	8 (53.3)	17 (28.4)
Factors influencing choice of health provider		
Convenience	10 (66.6)	27 (30.6)
Affordability	8 (53.3)	61 (69.3)
Family decision	5 (33.3)	40 (45.4)
Providers reputation	8 (53.3)	31 (35.2)
Confidentiality	2 (13.3)	15 (17.0)

## Discussion

This present study shows that lymphatic filariasis is endemic in Ado LGA of Benue State, Nigeria; with an overall hydrocoele prevalence of 8.5%, lymphoedema prevalence of 6.4% and CFA prevalence of 32.6%. Lymphatic filariasis due to *W. bancrofti* infections is indeed a serious public health problem in this area. This prevalence rate is higher than that of earlier observation in other parts of Benue State by (Omudu and Okafor, 2007) and parts of Nigeria by (Anosike et al., 2005; Anosike, 1994) in Bauchi State; in Cross River State (Braide et al., 2003; Udiodung et al., 2008); in Plateau State (Eigege et al., 2002) and in Anambra State (Mba and Njoku, 2000). However, prevalence of lymphoedema, hydrocoele and CFA in this area is relatively low when compared with findings from the Niger Delta area (Udonsi, 1986), Taraba State (Badaki and Akogun, 2000) and Kogi State (Nwoke et al., 2006).

The high endemicity of lymphatic filariasis in these communities could be due to several factors, especially the local environmental conditions like the availability of numerous domestic and peri-domestic mosquito breeding sites and deteriorating sanitary conditions. The various activities of the local population such as rice farming, cassava processing, fishing and other outdoor related activities tend to increase man-mosquito contact rates in different communities. Lymphatic filariasis vectors have been reported to breed in pots used for cassava fermentation (Udonsi, 1986; Iwuala, 1979). The topography of the area also created conducive environment for breeding of other vectors of filariases, such as clear, highly-oxygenated and fast flowing rivers like Okpokwu and Ado provide breeding grounds for black flies, (vector of onchocerciasis). This accounted for the cases of leopard skins, ochocercal nodules and onchodermatitis encountered during clinical examinations.

Age-related infection rates observed in this study agree with previous findings (Anosike et al., 2005; Eigege et al., 2002; Nwoke et al., 2006; Omudu and Okafor, 2007), which showed that prevalence of hydrocoele, lymphoedema and CFA increased with age. Apart from immunological reasons, duration of exposure to vectors in middle age and farming age group may be the major reason (Anosike et al., 2005; Eigege et al., 2002; Gyapong et al., 1998). The use of clinical manifestations of lymphatic filariasis as rapid assessment procedures for community diagnosis has been suggested (Eigege et al., 2002; Nwoke et al., 2006; Gyapong et al., 1998; Mwobabia et al., 2000). Our findings support the recommendations for the use of community lymphoedema and hydrocoele prevalence as rapid community indicator for lymphatic filariasis endemicity. Available parasitological and serological methods are either too cumbersome or too expensive (Anosike et al., 2005; Eigege et al., 2002; Omudu and Okafor, 2007; Weil et al., 1997).

On the communities' knowledge and beliefs in relation to lymphatic filariasis in the area, our findings revealed significant differences in lymphatic filariasis related knowledge between affected and unaffected respondents. This contrast with similar study in South India (Ramaiah et al., 1996) which reported that unaffected people, irrespective of their educational status, are more knowledgeable than the affected on the cause of filariasis. The general awareness of the cause transmission and prevention of the disease is poor; the role of mosquitoes in transmitting the parasitic agents of filariasis is poorly appreciated in many of the communities investigated. This study has demonstrated several other shortcomings in the communities' understanding of the disease. Our findings corroborate similar studies in Nigeria and elsewhere (Anosike et al., 2005; Braide et al., 2003; Ramaiah et al., 1996; Omudu and Okafor, 2008) and clearly underscored the importance of comprehensive community education to address identified gaps in perception and practices. The chronic manifestations of lymphatic filariasis can have significant, and often very negative social impacts. Clinical manifestations like lymphoedema of the limbs and external genitalia as seen in this area have a profoundly detrimental effect on the quality of life of affected individuals and their family members. The degree of social disability varies between cultural settings and prevailing community perceptions and practices, but the degree of stigmatization appear to be directly correlated with the severity of visible disease (Addiss and Brady, 2007); Person et al., 2006). There is significant evidence that patients experience stigma as a result of lymphatic filariasis in the communities investigated and this results from general community beliefs and perception on the causes and mode of transmission of the disease. While patients may experience withdrawal from social gathering, their family members could experience difficulty in finding desired spouses. Similar findings have also been reported in Ghana (Ahorlu et al., 1999), Sri-Lanka (Wijesinghe et al., 2007), Haiti (Person et al., 2006), Dominican Island (Person et al., 2006) and has been a subject of reviews (Hartigan 1999; Okafor and Omudu, 2005).

Unaffected respondents seem to underestimate lymphatic filariasis related health expenditures; the disease is known to cause direct and indirect economic loss to individuals and families. Studies from India, Ghana and Haiti indicate treatment costs to patients range from US $0.25 to US$2.62 per episode, as much as two days wages (Ramaiah et al., 1996, 1998). The cost of treatment included direct costs of treatment, including self mediation, as well as travel, feeding and accommodation since in most instances, the health providers live outside the patients' community. Our study demonstrates the need for the development of health education programmes that will enable people to protect themselves against mosquito bites. As Nigeria commence her lymphatic filariasis elimination programmes, there is an urgent need to develop morbidity management activities that will alleviate the burden of patients. Studies of this nature are critical to delineating lymphatic filariasis communities and needs to be replicated in other parts of the country where the status of the disease is unknown.

## Figures and Tables

**Table 3:** Respondents perception of the most worrisome signs and symptoms of lymphatic filariasis

	Affected n =15 Yes (%)	Unaffected n=88 Yes (%)
Fever	5 (33.3)	14 (15.9)
Chills	6 (40.0)	13 (14.7)
Pains	13 (86.6)	31 (35.2)
Swelling	15 (100.0)	50 (56.8)
Functional impairment	10 (66.6)	24 (27.2)
Appearance	11 (73.3)	37 (42.8)
Physical discomfort	9 (60.0)	46 (52.3)
